# Missing Aspirated Impacted Denture Requiring Tracheotomy for Removal

**Published:** 2017-11

**Authors:** Sampan-Singh Bist, Mahima Luthera, Poonam Arora, Lovneesh Kumar

**Affiliations:** 1 *Department of Otorhinolaryngology , Himalayan Institute of Medical Sciences, SRH University, Jolly-grant, Doiwala, Dehrdun 248140 (Uttarakhand) India. *; 2 *Department of Anaesthesia, Himalayan Institute of Medical Sciences, SRH University, Jolly-grant, Doiwala, Dehradun -248140 (Uttarakhand) India. *

**Keywords:** Denture, Foreign body, Tracheotomy

## Abstract

**Introduction:**

Aspirated foreign bodies continue to present challenges to otorhinolaryngologists. Removal of impacted airway foreign bodies via conventional methods can at times pose difficulty. This may be related to the location and type of foreign body, experience of the surgeon and anesthetist, and the availability of appropriate instruments. In adults, especially in edentulous patients, a swallowed denture usually gets lodged in the esophagus and entrance into the airway is uncommon.

**Case Report:**

We report a case of an uncommon foreign body (3-toothed artificial denture plate) impacted in the trachea of a 40-year-old male following an acute episode of an epileptic attack in which conventional methods of foreign body removal had failed. It was eventually removed via a direct laryngoscopy and tracheotomy technique.

**Conclusion:**

This type of impaction of a large denture in the trachea is uncommon and late presentation after aspiration is even more rare. This unusual case of a foreign body in the airway is interesting due to its rarity, mode of entry, site of impaction, variable clinical presentation, and method of removal; and hence, prompted the authors to report this case.

## Introduction

 The larynx is known as the watch dog of the airway since the lower respiratory tract is protected by the sphincteric action of the larynx and inhalation of a foreign body is extremely unusual in adults as opposed to swallowing (1). Denture as a foreign body in the esophagus is a more common occurrence in the adult population, whereas aspiration of a denture in the trachea is very rare and lack of symptoms is extremely rare. Full or partial dentures are used by approximately one in five people aged between 18-74 years (2). 

In developing countries such as India, foreign body denture is a growing concern, due to poor oral health and an increasing proportion of the elderly population (3). According to literature, maxillofacial trauma, dental treatment procedures, or ethanol intoxication and dementia are the main reasons for an aspirated tooth or denture (4). The accurate diagnosis may elude even the experienced physician due to the absence of typical initial choking incidents. Moreover, the delayed symptoms may mimic other common respiratory ailments like asthma, pneumonia or upper respiratory tract infections (5-7). 

Radiological diagnosis may be impeded by the presence of radiolucent acrylic resins in the dentures. Other denture materials such as porcelain and plastic artificial teeth are also difficult to visualize on plain radiographs (8). Once suspected, timely intervention to confirm the diagnosis followed by urgent removal of the aspirated foreign body is the key for the successful management and avoidance of complications in patients with airway foreign bodies. Accurate and early diagnosis can be achieved with associated history, physical examination, radiographic studies and direct laryngoscopy or a bronchoscopy. Delayed presentation may be associated with significant complications secondary to trauma and perforation of the upper aero-digestive tract. We report an unusual case of late presentation of an impacted denture in the trachea, without any acute respiratory symptoms and removed via an unusual method. 

## Case Report 

**Fig 1:**
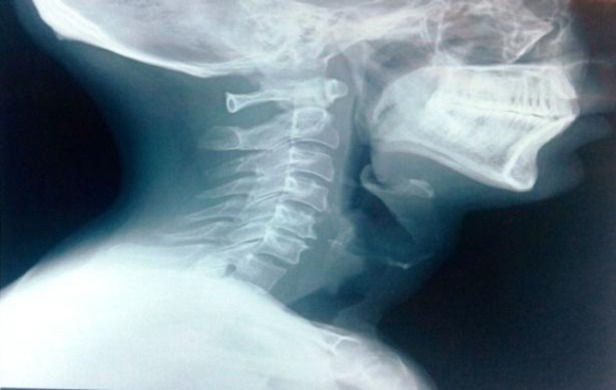
Lateral soft tissue neck radiograph demon- strating tracheal shadow containing vague opacity below the larynx.

A 40-year-old male presented to the ENT outdoor clinic with complaints of a foreign body sensation in the throat, progressive hoarseness of the voice and difficulty in breathing since the last 7 days. The patient developed the above mentioned symptoms after an acute episode of seizure. He also gave a history of five such episodes in the last two years, but he was not on any treatment for seizures. The patient was using an artificial denture for the upper jaw since one year. The patient became unconscious for 5-10 minutes after the last episode of seizure, after which he could not find his denture. He realized that he had accidentally lost his denture during the episode and ignored the whole event. Subsequently he had sudden onset of foreign body sensation in the throat and, later on, he developed progressive hoarseness and difficulty in breathing for which he consulted our hospital.  The patient complained of throat pain, painful swallowing and difficulty in speaking associated with discomfort in the throat.  There was no hemoptysis or chest pain. During clinical examination, the laryngeal contour was normal and crepitus was present. There was no stridor or chest indrawing. Examination of the oral cavity and oropharynx was normal. During auscultation, normal bilateral air entry was observed. A diagnostic laryngoscopy was performed, and revealed an inflamed edematous mucosa in the subglottic area with a whitish structure believed to be a foreign body (missing denture) or a growth in the subglottic region. The subglottic area had space to allow for breathing and vocal cords were mobile. A radiograph of the soft tissue of the neck (lateral view) was done, which showed a vague soft tissue shadow and narrowing of air columns (Fig.1). 

**Fig 2:**
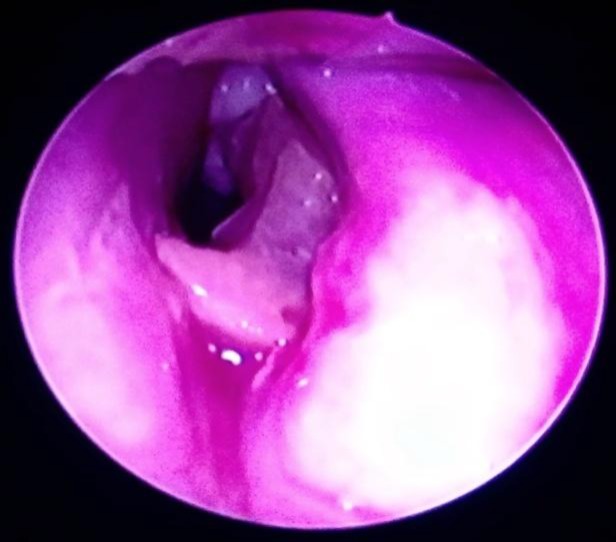
Endoscopic view showing impacted denture at the subglottis and trachea.

A direct laryngoscopy was planned and consent for rigid bronchoscopy and tracheostomy was obtained in view of a large and impacted denture.  General anesthesia was induced with Sevoflurane. Standard cardio- pulmonary monitoring and pulse-oximetry were used. Intubation was not possible with such a presentation. After induction, the patient was positioned and the laryngoscope was introduced. The denture was clearly observed to be impacted at the level of the subglottic region of the larynx and trachea (Fig.2).

**Fig 3:**
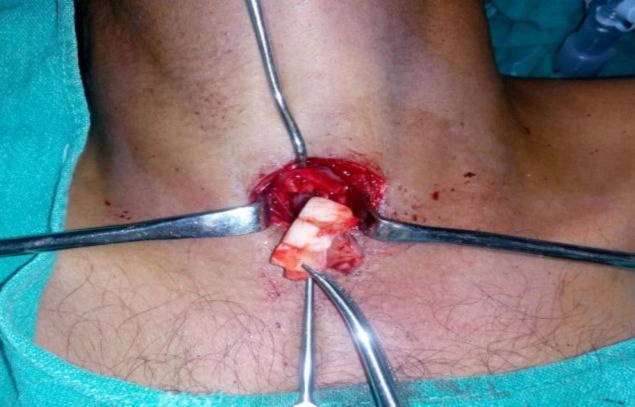
Denture removed via tracheotomy technique from the trachea.

**Fig 4:**
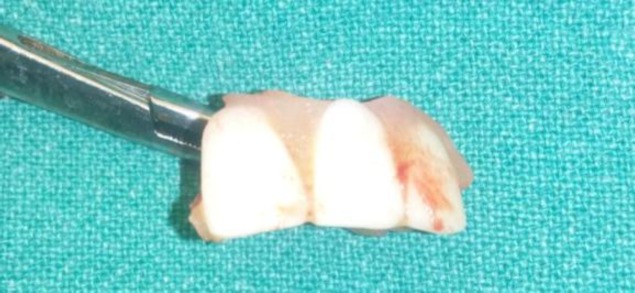
Impacted 3-toothed denture plate.

An attempt was made to remove the denture through the laryngoscope but was not succesful due to edema and impaction of the denture in subglottic area. Keeping in mind the possibility of injury to the vocal cords and nearby vital structures by this large irregular sharp denture, forceful removal was not attempted. A tracheotomy was performed. Direct laryngoscope was introduced and the foreign body was gently pushed from above by an assisting surgeon and gradually pulled through the tracheostome (Fig.3). An impacted 3-toothed denture plate was carefully removed by using curved artery forceps (Fig.4). 

Check bronchoscopy was performed and edema and mucosal injury was observed at the site where the foreign body was embedded. Spontaneous ventilation was maintained throughout the procedure.  Post-operatively, the patient was closely monitored. The patient was kept on nasogastric tube feeding for 2 days and tracheostomy was successfully closed on the 4^th^ day. A neurology consultation was performed for the seizure disorder and medical treatment was started. The patient was discharged on the 7^th^ post operative day. During his last follow up, at 8 weeks, he had a normal voice without any breathing problems.

## Discussion

Accidental inhalation of a foreign body has been reported in the literature (9), but a large impacted 3-toothed denture plate in the subglottis and trachea is quite unusual. The signs and symptoms due to foreign body aspiration can be described in three stages.  The patient presents with a history of a choking episode, along with paroxysms of gagging and coughing.  There may be no difficulty in breathing or there may be an acute respiratory obstruction demanding immediate attention. Once the patient gets over this phase, the second stage continues as an asymptomatic interval.  If not managed, the third stage is characterized by symptoms of complication, which are due to long-standing foreign body retention. These are related to either the delayed diagnosis or missed foreign bodies which initiate granulations. On many occasions, patients suffer from initial coughing paroxysms and only a mild respiratory distress.  A history of symptoms like coughing and/or choking or wheezing and unilateral reduced breath sounds (observed during examination) is almost a sure indicator of an inhaled foreign body. Usually the diagnosis of a foreign body in the trachea in the acute phase of entry is straightforward because of the readily available history of intake and signs and symptoms referable to the foreign body in the highly sensitive air passage (10). However, this may not be the case in an unconscious patient. The case reported here, dealt with an unconscious period during the seizure episode. In this case, initial symptoms were not evident due to the unconsciousness of the patient following the seizure episode. Apart from the missing artificial denture, he was not aware of the accidental inhalation of the denture. 

According to the patient, his denture was well fitted. Most likely, during the seizure episode, when the patient fell down, the denture was dislodged from the jaw and, due to a sharply indrawn breath, the foreign body would have been impacted in the subglottis and trachea. Although many elderly people wear dentures, its accidental inhalation is extremely rare and then its impaction at the level of the subglottis and trachea is even more rare.  Dentures are more often swallowed rather than inhaled (11). When a denture is inhaled, it can get stuck in the supraglottis or glottis and rarely in subglottis or trachea, such as in this case. But the aspiration of a large 3-toothed denture without any initial symptoms and presenting 7 days after the aspiration, was astonishing. It is remarkable that the patient did not have severe respiratory distress despite the presence of a large foreign body in the trachea. This may be explained by the fact that a foreign body can be lodged in the antero-posterior position without causing much respiratory distress. The development of progressive breathing difficulty and hoarseness in this case indicates that the denture was impacted in subglottic region. 

This caused disruption through repeated deglutition, which resulted in increased edema of the subglottis, which later on lead to increased symptoms in the patient. The degree of edema and granulation formation was in itself sufficient justification for tracheostomy which allowed easy removal of the foreign body and prevented the sudden complete closure of the airway. 

Diagnosis of denture and fragment of denture can be challenging as patients can present with a variety of symptoms and furthermore, radiolucency of dentures makes radiological diagnosis difficult. Thus, there is the need for the inclusion of a radio-opaque marker in acrylic based dentures or in denture production, as this may reduce the incidence of missed or delayed diagnoses. Rigid bronchoscopy is the best procedure to treat aspiration of a sharp foreign body which is a life threatening condition.  

A rigid bronchoscope allows better examination of the airway in patients with a longstanding retained foreign body. At times, a tracheostomy may be needed for the removal of the foreign body, such as in our case.  The patient did not suffer any complications despite having delayed presentation. The rescue of the foreign body was facilitated by technical improvements with the rod lens telescope, video endoscopy, a broad range of a variety of sized forceps, and safe anesthesia. In spite of these advances, more than 3000 documented deaths occur per year because of foreign bodies and an untold number of patients survive with variable sequelae (5,6). 

The average lifespan of complete dentures is about 5–7 years and wearing out of these dentures is a result of poor fitting rather than a mechanical failure. 

Many elderly people wear dentures and therefore they need to be properly educated about the care of these dentures to avoid ingestion or inhalation. Therefore, it is important to discourage patients from wearing them during sleep and from using either ill-fitting or damaged dentures. In all patients with a broken or swallowed denture, there is always a chance of inhalation of the denture in part or as a whole.

## Conclusion

A foreign body in the airway can also present without any obvious symptoms of aspiration in the initial stage. However, a high index of suspicion is highly imperative when a patient presents with a history of a missing denture, even if the patient does not have symptoms of respiratory obstruction. Rigid bronchoscopy is considered the treatment of choice for removal of the majority of foreign bodies from airways. However, sometimes, tracheotomy is a safe method for removal of a large impacted foreign body.  
